# Supplementary material for: Closing the gender gap in competitiveness through priming
Source: Nat Commun. 2018 Oct 19;9:4359. doi: 10.1038/s41467-018-06896-6 (PMC6195557; doi:10.1038/s41467-018-06896-6)
Supplement: Supplementary file 1 — Supplementary Information [file 41467_2018_6896_MOESM1_ESM.pdf]

Supplementary Information for:

CLOSING THE GENDER GAP IN COMPETITIVENESS THROUGH PRIMING

Balafoutas et al.

Supplementary Methods

1. Experimental instructions

This section contains the instructions that subjects received in the experiment. The instructions are translated from German.

PRIMING

Notes to PRIMING:

Within each session, some participants were assigned to the high- and others to the low prime condition. However, all participants were unaware of the power prime manipulation that others in their session received and only read the information regarding the writing task that they were supposed to complete.

Instructions for PRIMING:

Text for High priming condition

Please recall a particular incident in which you had power over another individual or individuals.

By power, we mean a situation in which you controlled the ability of another person or persons to get something they wanted, or were in a position to evaluate those individuals.

Please describe this situation in which you had power — what happened, how you felt, etc.

Text for Low priming condition

Please recall a particular incident in which someone else had power over you.

By power, we mean a situation in which someone had control over your ability to get something you wanted, or was in a position to evaluate you.

Please describe this situation in which you did not have power — what happened, how you felt, etc.

You have 10 minutes for this task.

How much power, on a scale from 1 to 9, did you have in this situation? Where 1 = absolutely powerless and 9 = absolutely powerful. (Please mark the applicable number)

1 2 3 4 5 6 7 8 9

COMPETITION EXPERIMENT

Notes to COMPETITION EXPERIMENT:

The experiment consisted of three stages. The instructions for each stage were handed out after completion of the previous stage. After completion of Stage 1, participants were asked to indicate their belief about how well they ranked within their session, and in particular in which quartile (top, second, third, or fourth). After completion of Stage 2, participants were asked to indicate their belief about how well they ranked within their group (first, second, third, or fourth). Correct beliefs were rewarded with €1 each.

Instructions for COMPETITION EXPERIMENT:

<u>Stage 1: Piece rate payment</u>

Your task in Stage 1 is to solve correctly as many addition exercises as possible. To be more precise, you will have 3 minutes time in order to solve as many additions of five randomly selected two-digit numbers as possible, by entering the sum of the five numbers. You are not allowed to use calculators but you can write down the numbers and use the provided scrap paper for your calculations. You enter an answer by clicking with the mouse on the "Confirm" button. When you enter an answer, you immediately find out on the screen whether it was correct or not.

If Stage 1 is the stage selected for payment (among Stages 1-3), then you will receive **€1.00 for each correct answer** that you entered within the 3 minutes. Your payment is not reduced when you enter a wrong answer.

Directly before the start of this stage you will be given one minute in order to familiarize yourselves with the screen: During this time, you can solve addition exercises, which do not count for the experiment. Afterwards, Stage 1 will begin.

<u>Stage 2: Tournament payment</u>

As in stage 1, you will have 3 minutes time in order to solve correctly as many addition exercises as possible. However, your payment in this stage depends on your performance relative to the performance of a group of participants.

Group composition: Each group consists of 4 participants, 2 of whom are men and 2 are women. Groups are randomly formed at the beginning of this stage and **each participant stays in the same group until the end of this part of the experiment**.

If Stage 2 is the stage selected for payment (amongst Stages 1-3), then your payment depends on how many additions you have solved correctly in comparison with the other three participants in your group.

The group member who has entered the most correct answers is the winner of the tournament. The winner receives **€4.00 per correct answer** each, while the other three members **do not receive any payment**. In case of a tie, the ranking among the members with equal performances is determined randomly. You will not be informed about the outcome of the tournament until the end of the experiment.

Stage 3: Choice between Piece rate payment and Tournament payment

As in Stages 1 and 2, you will have 3 minutes in order to solve correctly as many addition exercises as possible. However, you must now choose your preferred payment method for your performance in Stage 3. You can either choose a Piece-rate payment (as in Stage 1) or a Tournament payment (as in Stage 2).

If you choose the **Piece rate payment**, then you will receive **€1.00 per correct answer.**

If you choose the **Tournament payment**, then your performance in Stage 3 will be evaluated in comparison to the performance of the other three group members **in Stage 2**. As a reminder: That is the stage that you have just completed.

If you enter more correct answers than all three of your group members did in Stage 2, then you will receive **€2.00 per correct answer.** In other words, no group member of your group can have a Stage 2-performance which is higher than your Stage 3-performance, otherwise you receive **no payment** for this stage.

In case of a tie, the ranking among the members with equal performances is again determined randomly. The group composition (with 2 men and 2 women) is as it was in Stage 2. If you choose the Tournament payment, you will not be informed about the outcome of the tournament until the end of the experiment.

2. Elicitation of risk attitudes in the Investment Game

In order to elicit risk attitudes, we administered after the end of Stage 3 (Competitive Task) a simple investment game [1]. Participants received an endowment of 12 experimental currency units (ECUs; 1 ECU = €0.25), which they could invest in a risky asset, or keep for themselves. Any amount between 0 and 12 ECUs could be invested. The investment had a 50% chance of being successful. In case of success, the invested amount was multiplied by 2.5 and paid back to the participant. If the investment was not successful, the invested amount was lost. ECUs not invested were directly kept by participants. Higher investments in this task thus indicate a greater willingness to take risks.

Investment rates are shown in Supplementary Figure 1 and discussed in the Notes to Supplementary Table 4.

Supplementary Figures

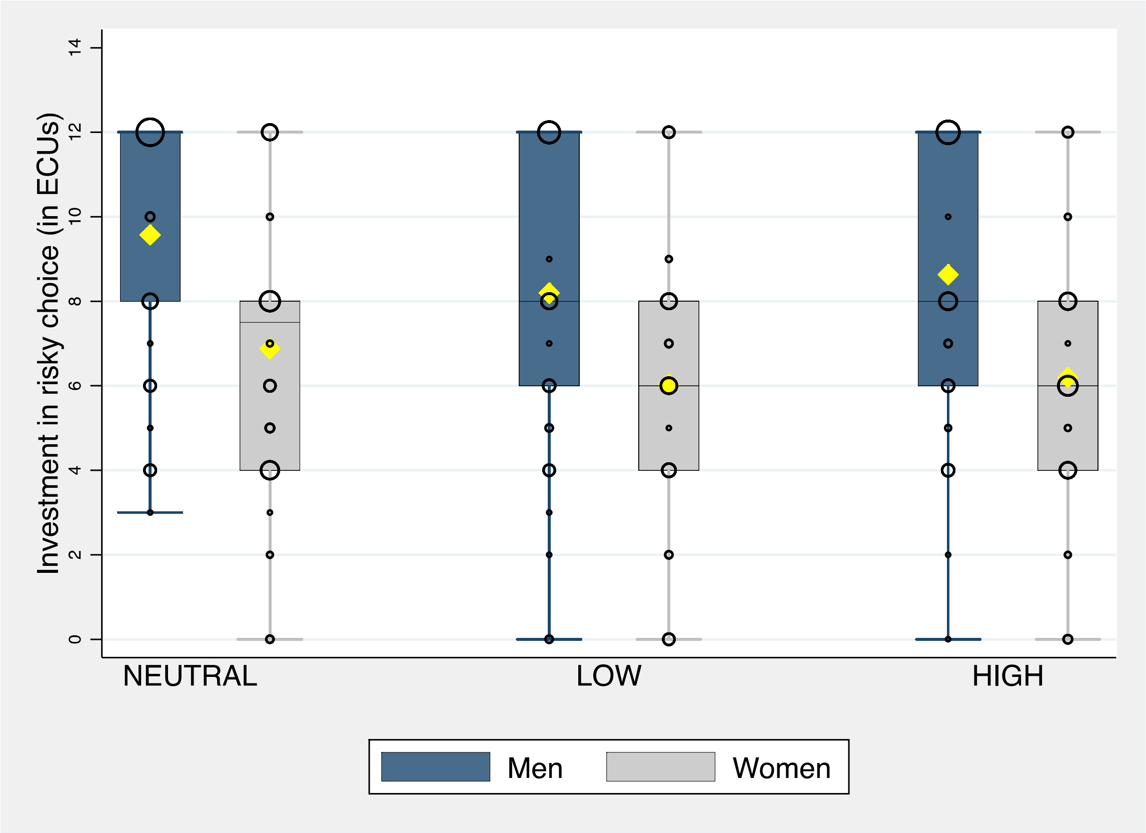

Supplementary Figure 1. Amount of invested ECUs (min = 0, max = 12) in the investment game, by priming condition and gender (N=401). Box plots show the mean (indicated by yellow diamond signs), the 25th and 75th percentiles, Tukey whiskers (median ± 1.5 times interquartile range), and individual data points. Larger dots indicate a higher number of participants with the corresponding invested amount.

Supplementary Tables

Supplementary Table 1. Regression analysis of the reported power scale.

Dep. Variable:	power scale
female	-0.05<br>(0.35)
HIGH	2.73^{***}<br>(0.34)
female x HIGH	0.18<br>(0.48)
constant	3.44^{***}<br>(0.24)
N	256

Ordinary least squares regressions. Standard errors in parentheses.

*** $P < 0.01$. One subject did not report the power scale.

Notes Supplementary Table 1:

The results in Supplementary Table 1 show a significant effect for HIGH (coefficient = 2.73, t = 8.39, P < 0.001, N = 256), indicating that – as expected – high power primed participants reported a significantly higher power scale compared to LOW. This effect is independent of gender, as evinced by the insignificant interaction term *female x HIGH*.

Supplementary Table 2. Mean responses to the mood questionnaire, by treatment. Standard deviations in parentheses.

	NEUTRAL	LOW	HIGH	NEUTRAL vs. LOW	NEUTRAL vs. HIGH
Good-Bad Mood (GB)	4.01 (2.79)	3.04 (3.27)	2.84 (3.03)	P = 0.011	P < 0.001
Awake-Tired Mood (AT)	1.04 (3.57)	-0.37 (3.66)	0.13 (3.69)	P = 0.002	P = 0.033
Calm-Nervous Mood (CN)	3.20 (2.97)	3.15 (3.30)	3.39 (2.99)	P = 0.853	P = 0.512

Notes Supplementary Table 2:

Responses in each dimension range from 1 to 5, with higher values indicating a better (more awake, calmer) mood. P values correspond to Mann-Whitney tests.

The table reveals that mood is not evenly balanced across treatment. Specifically, the GB and the AT dimension differ significantly across priming condition: participants are on average significantly more tired and in a worse mood in LOW and HIGH compared to NEUTRAL. Disaggregating by gender, we find that HIGH leads to a worse mood on average for both genders (differences between NEUTRAL and HIGH in the GB dimension: Mann-Whitney U tests, $z = 1.90$, $P = 0.057$, $N = 140$ for men; $z = 3.09$, $P = 0.002$, $N = 138$ for women,), and to a more tired state for women (Mann-Whitney U, $z = 2.05$, $P = 0.040$, $N = 138$). Given the fact that the mood variables are not independent of priming condition, we omit them from the subsequent analysis on competition entry decisions in order to avoid issues of endogeneity.

Supplementary Table 3. Overview of experimental participants by gender and priming condition.

Priming	Men	Women	Total
NEUTRAL	72	72	144
LOW	63	60	123
HIGH	68	66	134
Total	203	198	401

Supplementary Table 4. Regression analysis of competition entry decisions.

	(1)	(2)	(3)
Dep. Variable	competition	competition	competition
female	-0.84*** (0.24)	-0.67*** (0.26)	0.45* (0.27)
LOW	-0.06 (0.22)	0.05 (0.24)	0.18 (0.24)
HIGH	-0.34 (0.22)	-0.39* (0.24)	-0.30 (0.25)
female x HIGH	0.57* (0.34)	0.73** (0.37)	0.76** (0.38)
female x LOW	0.30 (0.34)	0.29 (0.37)	0.26 (0.38)
performance Stage 1		0.15*** (0.05)	0.14** (0.05)
performance Stage 2		-0.11** (0.05)	-0.10* (0.06)
belief Stage 1		-0.12 (0.11)	-0.14 (0.11)
belief Stage 2		-0.67*** (0.13)	-0.59*** (0.14)
risk tolerance			0.11*** (0.03)
age		0.01 (0.01)	0.02 (0.01)
constant	-0.25* (0.15)	0.78 (0.57)	-0.49 (0.66)
N	401	401	401

Probit regressions. Standard errors in parentheses. * $P < 0.10$, ** $P < 0.05$, *** $P < 0.01$. Dependent variable: competition (0 = decided against competition in Stage 3, 1 = decided for competition in Stage 3). *belief Stage 1*: "I think I performed at: 1=best 25%, 2=best 26-50%, 3=best 51-75%, 4=lowest 25% of the participants in this session". *belief Stage 2*: "I think I

was the 1=best, 2=second best, 3=third best, 4=fourth best performer within my group”. *risk tolerance* from 0 to 12, where higher values indicate lower risk aversion.

Supplementary Table 5. Mean invested amounts, by gender and priming condition (standard deviations in parentheses).

Priming	Men	Women	Total
NEUTRAL	9.57 (3.00)	6.88 (3.25)	8.22 (3.40)
LOW	8.19 (3.65)	6.03 (3.36)	7.14 (3.66)
HIGH	8.63 (3.25)	6.20 (2.88)	7.43 (3.30)
Total	8.83 (3.33)	6.39 (3.17)	7.63 (3.47)

Notes Supplementary Table 5:

Investment rates in Supplementary Table 5 reveal that women invested significantly less than men on average ($P < 0.001$, $N = 401$). This is true independently of priming condition (NEUTRAL: $P < 0.001$, $N = 144$; LOW: $P = 0.002$, $N = 123$; HIGH: $P < 0.001$, $N = 134$).

Besides gender, treatment affects risk attitudes: investment rates are overall lower in LOW and HIGH compared to NEUTRAL ($P = 0.026$, $N = 267$; $P = 0.059$, $N = 278$, respectively). Disaggregating by gender, there is no significant difference between LOW and HIGH for any of the two genders ($P > 0.563$). Comparing NEUTRAL to LOW, investment rates differ for men ($P = 0.033$, $N = 135$) but not for women ($P = 0.229$, $N = 132$). Similarly, men invest significantly less in HIGH than in NEUTRAL ($P = 0.094$, $N = 140$), while this is not the case for women ($P = 0.263$, $N = 138$). All P values refer to Mann-Whitney U tests.

Supplementary Table 6. Average earnings (in €) in Stage 3, by gender and priming condition (N = 401). Standard deviations in parentheses.

	Men	Women	Both
NEUTRAL	7.47 (7.52)	6.44 (4.15)	6.96 (6.07)
LOW	6.02 (5.73)	6.68 (6.18)	6.34 (5.94)
HIGH	7.04 (6.39)	5.77 (4.61)	6.42 (5.60)

Notes Supplementary Table 6:

The table presents earnings in Stage 3, by gender and priming condition. The differences across the three treatments are insignificant for men (Kruskal Wallis test, $\chi^2 = 0.736$, P = 0.692, N = 203; Mann-Whitney U tests, P > 0.425 for three pairwise comparisons, N = 203) as well as for women (Kruskal Wallis test, $\chi^2 = 1.556$, P = 0.459, N = 198; Mann-Whitney U tests, P > 0.203 for three pairwise comparisons, N = 198). The same is true in the pooled sample, i.e., considering both genders (Kruskal Wallis test, $\chi^2 = 0.801$, P = 0.670, N = 401; Mann-Whitney U tests, P > 0.420 for three pairwise comparisons, N = 401).

Supplementary Table 7. Regression results on earnings in Stage 3, by gender

	Men	Women
LOW	-0.31	0.74
	(0.93)	(0.65)
HIGH	0.39	-0.05
	(0.91)	(0.63)
performance Stage 3	1.28^{***}	1.24^{***}
	(0.12)	(0.10)
constant	-2.66^{**}	-2.25^{***}
	(1.16)	(0.81)
N	203	198

Notes: Dependent variable: Earnings in Stage 3. Ordinary least squares regressions. Standard errors in parentheses. * p < 0.10, ** p < 0.05, *** p < 0.01

Notes Supplementary Table 7:

The regressions in this table examine whether the priming intervention in treatments HIGH and LOW has an effect on the earnings of men and women in Stage 3. The two treatment dummies (HIGH and LOW) are insignificant for both genders, while performance in Stage 3 has the expected positive and highly significant coefficient.
